# Increase in Hospital Admissions for Severe Influenza A/B among Travelers on Cruise Ships to Alaska, 2015

**DOI:** 10.3201/eid2403.171378

**Published:** 2018-03

**Authors:** Michael Payne, Danuta Skowronski, Suzana Sabaiduc, Linda Merrick, Christopher Lowe

**Affiliations:** Providence Health Care, Vancouver, British Columbia, Canada (M. Payne, L. Merrick, C. Lowe);; University of British Columbia, Vancouver (M. Payne, D. Skowronski, C. Lowe);; BC Centre for Disease Control Public Health Laboratory, Vancouver (D. Skowronski, S. Sabaiduc)

**Keywords:** influenza, H3N2, travel, cruise ships, oseltamivir, Alaska, Vancouver, British Columbia, viruses, respiratory infections

## Abstract

An increase in hospital admissions for influenza occurred during the summer of 2015 at an acute care facility in Vancouver, British Columbia, Canada. Investigation identified 25 patients with recent history of cruise ship travel to Alaska. All characterized influenza A viruses were A(H3N2). We describe patient treatment regimens and outcomes.

Disorders of the respiratory tract are the most common cause of medically attended illness (29%) among cruise ship passengers and crew (*1*). Influenza outbreaks associated with travel on cruise ships, including those sailing to Alaska in the summer months (*2*–*5*), have been described previously. The number of travelers on cruises to Alaska is large, with ≈900,000 documented in 2011 (*6*). We report an increase in hospital admissions for severe influenza among travelers on cruise ships to Alaska during the summer of 2015 at an acute care facility in Vancouver, British Columbia, Canada.

## The Study

During the 2015 summer season (June–September), an increase in admissions for severe influenza was detected at our hospital, despite minimal influenza activity in the community (*7*,*8*). We determined that 25 cruise ship travelers to Alaska tested positive by PCR for influenza at hospital admission, 24 for influenza A and 1 for influenza B. We included all 25 of these patients in our study. We reviewed patient charts to determine date of illness onset, vaccination status, risk factors, treatment with antivirals or antibiotics, and patient outcomes (*9*). We sent influenza A–positive samples to the provincial public health reference laboratory for typing and performed gene sequencing on an additional subset to establish phylogenetic clade. 

The study group consisted of 11 women and 14 men (average age 72.5 years). Twenty-two travelers were from the United States, 2 from Canada, and 1 from the United Kingdom. Only 4 patients had receipt of the 2014–15 influenza vaccine documented in their medical chart. All patients had >1 risk factor for severe influenza, with an average of 2.5 recognized risk factors per patient. The most common were age >65 years (84%), cardiovascular disease (48%), and pulmonary disease (44%). Symptom onset occurred on average 4.1 days before admission. Eight patients were documented to have an earlier influenza diagnostic test performed on board the ship; 7 were positive, and 1 was negative. All 7 of these influenza-positive patients were started on oseltamivir before hospital admission. However, none of the patients who were negative or not tested for influenza on the cruise ship were documented to have been empirically treated with oseltamivir. Before admission, antibiotics were prescribed to 12 patients, with an average course lasting 2.2 days.

Admission blood culture results were negative for all 25 patients. Admission sputum cultures were collected from 17 patients: 1 showed heavy growth of methicillin-resistant *Staphylococcus aureus*, and the other 16 showed no growth or normal respiratory flora. During hospitalization, oseltamivir treatment was administered to 19 patients, with average duration (including discharge prescriptions) of 6.1 days. Twenty-two patients were empirically started on antibiotics at hospital admission. The average length of antibiotic treatment, including discharge prescriptions, was 7.4 days from the date of admission.

In-hospital complications included 1 case of *Pseudomonas aeruginosa* ventilator-associated pneumonia, 1 myocardial infarction, and 1 tracheostomy. No in-hospital deaths occurred. The average length of hospitalization was 7.1 days. Patients with influenza diagnosed on the cruise ship and started on oseltamivir (n = 7) had an average length of stay of 5.3 days versus 7.8 days for patients with influenza diagnosed in hospital only (n = 18), but this difference was not statistically significant (p = 0.42 by 2-tailed *t*-test).

All influenza A virus samples were sent to a reference laboratory for subtyping and were identified as H3N2. In addition, 16/24 isolates underwent gene sequencing and were identified as clade 3C.2a, the dominant genetic and antigenic drift variant responsible for the 2014–15 influenza epidemic in the Northern Hemisphere. Clade 3C.2a viruses are distinguished from the 2014–15 A/Texas/50/2012 (clade 3C.1) vaccine strain by multiple (10–12) amino acid substitutions at antigenic sites of the surface hemagglutinin protein, notably F159Y and adjacent K160T mutations within immunodominant antigenic site B, the latter conferring a potential gain of glycosylation further relevant to antibody binding (*10*,*11*). The antigenic drift of clade 3C.2a viruses and their mismatch to the 2014–15 vaccine were widely recognized that season, with historically low vaccine effectiveness reported and recommendations issued for adjunct protective measures (*11*).

## Conclusions

We present a review of 25 cases of laboratory-confirmed influenza illness requiring hospital admission during the summer of 2015 and associated with cruise ship travel to Alaska. The study was initiated in response to the perception by hospital staff of an atypical surge in admissions for severe influenza in summer 2015, with 27 of 33 influenza A–positive patients having illness associated with cruise ship travel, compared with 1 of 3 cases in summer 2014 and 8 of 19 cases in summer 2016. 

The US Centers for Disease Control and Prevention (CDC) has published guidelines for the management of influenza-like illness (ILI) on cruise ships (*12*). For prevention, CDC recommends that crew and passengers receive the season’s recommended influenza vaccination, postpone travel when ill, and comply with respiratory etiquette and that persons who are ill be appropriately isolated. In addition, CDC recommends antiviral treatment in cases of confirmed or suspected influenza in ILI patients with severe manifestations or risk factors for severe disease. Cruise ship passengers, particularly those at high risk for severe influenza complications, should be advised of these recommended measures before travel to mitigate their risk.

In our review, 7 patients had influenza A diagnosed on the cruise ship; 1 patient had tested negative for influenza A/B on the ship. Only patients who tested positive for influenza A on the cruise ship were documented to have been started on oseltamivir; however, a positive test is not required for initiation of treatment, as per CDC guidelines, especially in the context of a documented outbreak (*12*). Point-of-care influenza A/B antigen tests have poor sensitivity; therefore, in the appropriate clinical scenario, treatment should not be withheld on the basis of a negative test (*13*). In this small case series, patients who received treatment with oseltamivir before admission had a shorter duration of stay (Δ = 2 days), although this difference was not statistically significant. Definitive conclusions cannot be drawn from this observation, although it corresponds with a previous metaanalysis that noted a 21% decrease in the time to alleviation of symptoms in the oseltamivir treatment versus placebo group (*14*).

Our case series has several limitations. First, minimal follow-up information was available after discharge from hospital. Second, all of our patients had countries of origin in the Northern Hemisphere, but propagated viruses might have been introduced from elsewhere. Cruise ships to Alaska include travelers from the Southern Hemisphere, where influenza peaks during June–September. Third, we relied on the cruise ship medical records to provide information on treatment before admission. For many patients, this information was not provided, and if so, we assumed that no treatment or further diagnostics occurred. Finally, our review focused on a single facility; patients admitted to other facilities in the region would add to the total disease burden but were not captured in this study.

In summary, we report a series of severe influenza cases requiring hospitalization among cruise ship travelers to Alaska during the summer of 2015. Cruise ship passengers should be advised of such influenza risks and preventetive measures before travel. Our findings reinforce the need for surveillance monitoring to inform timely initiation of antiviral treatment during cruise ship outbreaks. Clinicians caring for passengers with ILI should consider empiric influenza therapy, particularly because cruise ship travelers can include a large proportion of persons at risk of severe influenza complications.
